# Nucleation and Formation of a Primary Clot in Insect Blood

**DOI:** 10.1038/s41598-019-40129-0

**Published:** 2019-03-05

**Authors:** Pavel Aprelev, Terri F. Bruce, Charles E. Beard, Peter H. Adler, Konstantin G. Kornev

**Affiliations:** 1 Department of Materials Science and Engineering, Clemson University, Clemson, South Carolina 29634 USA; 2 Light Imaging Facility, Clemson University, Clemson, South Carolina 29634 USA; 3 Department of Plant and Environmental Sciences, Clemson University, Clemson, South Carolina 29634 USA

## Abstract

Blood clotting at wound sites is critical for preventing blood loss and invasion by microorganisms in multicellular animals, especially small insects vulnerable to dehydration. The mechanistic reaction of the clot is the first step in providing scaffolding for the formation of new epithelial and cuticular tissue. The clot, therefore, requires special materials properties. We have developed and used nano-rheological magnetic rotational spectroscopy with nanorods to quantitatively study nucleation of cell aggregates that occurs within fractions of a second. Using larvae of *Manduca sexta*, we discovered that clot nucleation is a two-step process whereby cell aggregation is the time-limiting step followed by rigidification of the aggregate. Clot nucleation and transformation of viscous blood into a visco-elastic aggregate happens in a few minutes, which is hundreds of times faster than wound plugging and scab formation. This discovery sets a time scale for insect clotting phenomena, establishing a materials metric for the kinetics of biochemical reaction cascades. Combined with biochemical and biomolecular studies, these discoveries can help design fast-working thickeners for vertebrate blood, including human blood, based on clotting principles of insect blood.

## Introduction

Living in an environment surrounded by natural enemies and microbial pathogens, insects have evolved distinct strategies to deal with wounding and potential infection^1–7^. The strategies associated with insect blood, such as thickening, typically operate at the molecular, nanometer, and microscopic scales. The phenoloxidase cascade^8,9^ was identified as the main molecular pathway in the response of insects to wounding and microbial attack, engaging a complex assembly of proteins, particularly the enzyme phenoloxidase, to thicken the blood^10^. Catalysis of phenoloxidase results in formation of a variety of immune defense molecules, high molecular weight macromolecular complexes, and pigments of the melanin group^11,12^. These molecules and macromolecular complexes trigger an orchestrated action of physico-chemical phenomena at different temporal and spatial scales to form a fibrous biopolymer gel with entrapped cells. Phenoloxidase is involved in cross-linking and melanizing the primary soft clot to form a solid, melanized clot^8,9^. The mechanisms and kinetics of nucleation and formation of a primary soft clot, however, remain enigmatic.

The coagulation cascade in insects happens markedly faster than that of vertebrates^13,14^. Yet, quantitative metrics of the phenomena have not been established. The most detailed analysis and classification of physical phenomena and patterns of cell aggregation associated with blood coagulation and clotting in insects was described in the last century^1,15–17^. Recent progress in understanding wound healing has been achieved with advances in microscopy and genetics, revealing that the cellular responses are coordinated by multiple signals from the wound^4,7,9,18,19^. Plugging of the wound, scab formation, and epidermal cell mobilization at wounds of *Drosophila melanogaster* happen at time scales of one hour and longer and operate under separate genetic controls^18,19^. Static measurements after one hour of incubation reveal that plasmatocytes spread across foreign substrates and granular cells attach to these plasmatocytes in large numbers, forming clusters^20^. To make the cells adherent, reversible molecular rearrangements occur to form clusters of adherent receptors on the surfaces of granular cells^21^. Labeling of these receptors suggests that the hemocytes become increasingly adhesive for the first 20 minutes after exposure to a foreign surface (Fig. 4B in^21^)

Hemolymph of some species, when leaking from the wound, triggers humoral reactions that cause self-assembly of lipids and proteins and lead to formation of fibrin-like threads^22,23^. Formation of these threads and gel-like structures relies on the use of modules at the protein level, forming different domains (some of which are glycosylated), and at the level of functional modules, such as phenoloxidase, transglutaminase, and different classes of clotting factors^1–7,17,24–26^.

In larvae of some insects, such as *Manduca sexta*, when the cell density is high, humoral reactions in hemolymph plasma and cell aggregation are orchestrated, demonstrating a collective behavior^1,15–17^. The physical features of blood cell aggregation at short time scales of minutes or less have never been detailed. At these short time scales, the physical and materials characteristics of clot formation have been mostly descriptive and poorly understood. We aim to fill this knowledge gap by providing a quantitative analysis of cell aggregation and by proposing physical mechanisms of blood thickening. The cell-rich blood of fifth-instar larvae of *Manduca sexta* was chosen as a model system^3,4,27–29^. Our primary goal was to identify the pattern of cell aggregation and quantify its kinetics at short time scales (minutes). To accomplish this objective, we developed and used nano-rheological magnetic rotational spectroscopy with nanorods. We show that cell aggregation occurs in two steps within several minutes: 1) the cells first gather into an aggregate, followed by 2) densification and rigidification of the aggregate, leading to formation of an elastic clot.

## Results and Discussion

**Figure 1:**
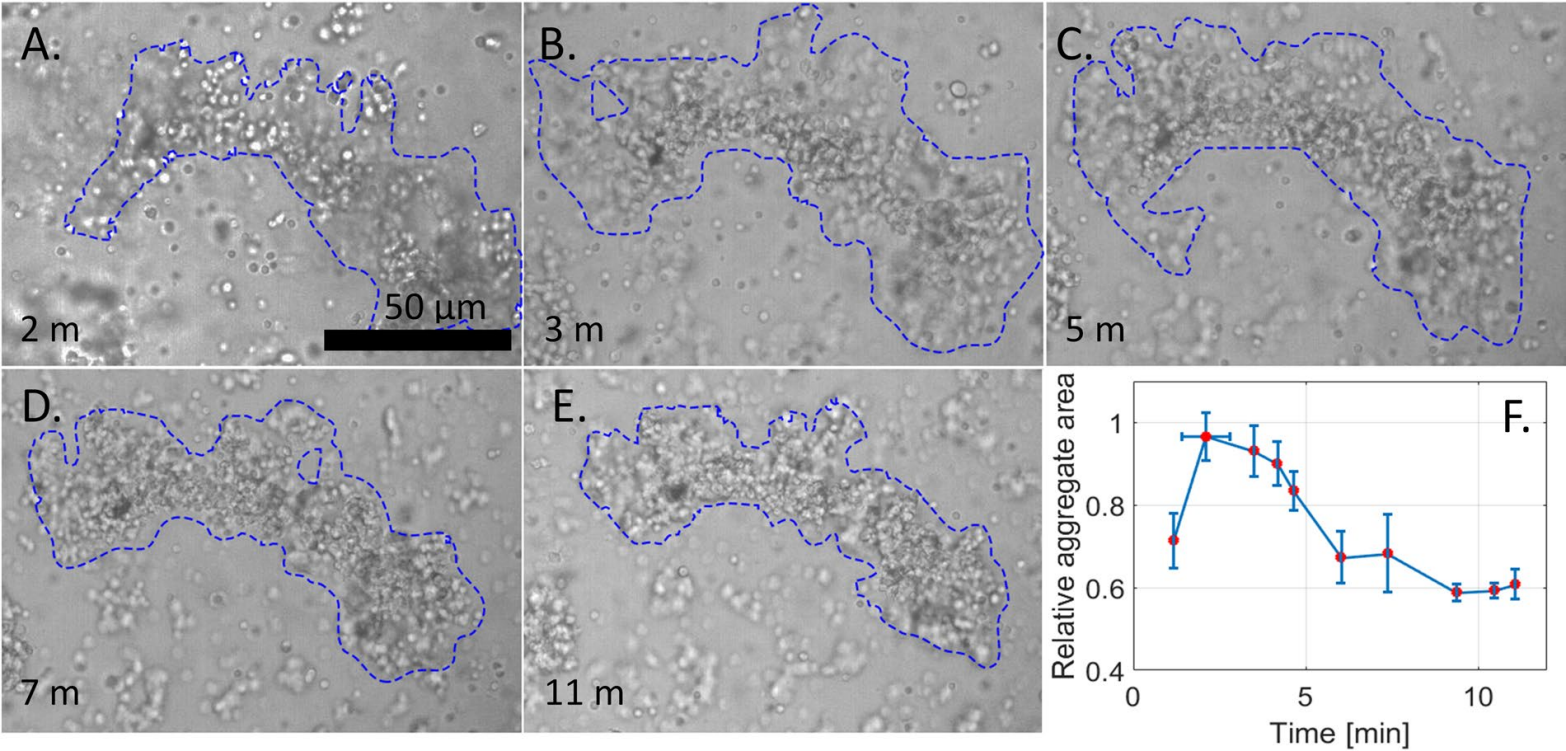
Growth and shrinkage of cellular aggregates in incubating hemolymph. (**A**–**E**) A gallery of bright-field micrographs of an *in-vitro* incubating droplet of blood from a larva of *M. sexta*. Cells can be seen as bright and dark circles freely floating in the fluid (grey background) and as part of the aggregate (blue outline); t is the incubation time of the sample. The boundary of the aggregate was determined using a Photoshop user-assisted image analysis algorithm, Quick Selection Tool. (**F**) Average non-dimensional area of aggregates of three larvae as a function of incubation time, t. The area was normalized by its maximum for each aggregate. The rate of area shrinkage approaches 0 after about 10 minutes of incubation.

Optical observations with bright-field microscopy of *in vitro* blood from three final-instar larvae of *Manduca sexta* revealed that blood cells assemble into larger aggregates within about 10 minutes. Tracking of blood aggregates in the bulk is problematic, as they continually move. Hence, quantitative analysis of aggregate growth was accomplished by focusing the objective on the glass substrate to which some cells adhered^15,17^ (Fig. 1). At *t* = 2 minutes, the aggregates were smaller than at *t* = 2.9 minutes. After 2.9 minutes, the average area of the aggregates began to decrease, initially at a high rate but then more slowly until 10 minutes.

At the time scale of these observations, individual cells in the aggregates neither grew nor multiplied. After 10 minutes, all free cells in the hemolymph had accumulated into aggregates. The aggregates were isolated and no physical communication between them was detected; once aggregates formed, no free cells joined them (see movie in Supplementary Material). Although initially loosely packed and branched, the aggregates became denser and less branched with time.

These observations confirmed a general pattern of blood aggregation in diverse insect taxa, as reviewed by Gregoire^17^. This general pattern allowed us to propose a working physical two-step scenario for the assembly of cells into aggregates, which aligns with previous microscopic observations and biomolecular and biochemical studies of cell aggregation at longer time scales (reviews by Gregoire^17^ and Strand^4^). In the first few minutes (*t* < 3*min*), the cells assembled into aggregates. Following the initial formation (3*min* < *t* < 10*min*), the aggregates densified and a polymer network grew that displaced the watery fluid from the aggregate. This second step in cell aggregation has been intensively studied by molecular and cell biologists, who have identified the role of different cells and biomolecular triggers that produce a polymer network^3–5,7,14^.

**Figure 2:**
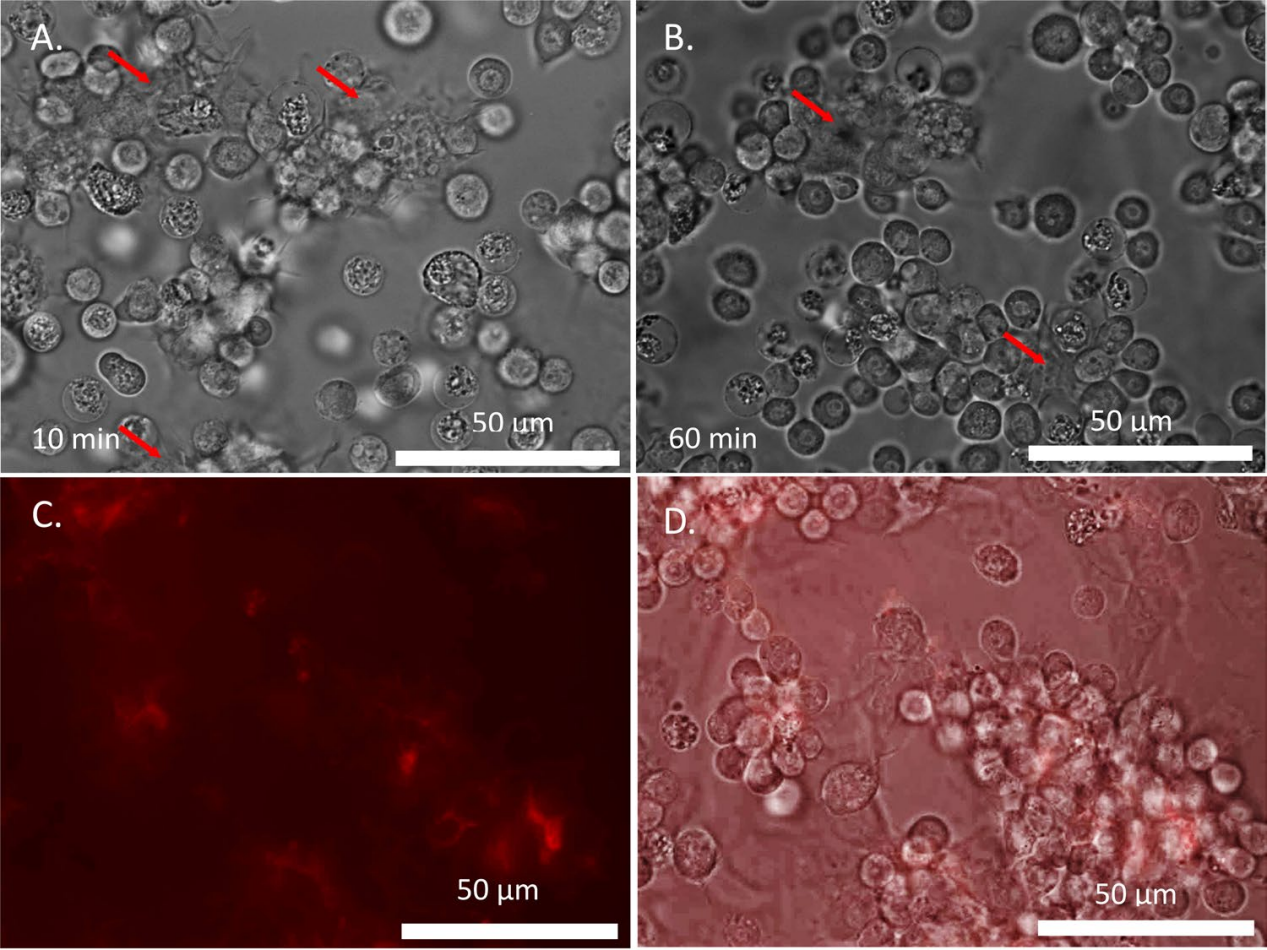
Micrographs of incubated hemolymph aggregates. (**A**,**B**) Time-lapse snapshots taken in the same location at t = 2 minutes (**A**) and t = 7 minutes (**B**) after extraction under an inverted transmitted-light phase-enhanced microscope. Aggregation of cells connected with an amorphous dark grey material is visible (red arrow). As incubation time increases, the aggregates become more closely packed and the grey material darkens. The average distance between adjacent cells at t = 2 minutes is 8.9 ± 1.5 μm (N = 27) and at t = 7 minutes is 7.6 ± 1.1 μm (N = 37). (**C**,**D**) Fluorescent micrograph of hemolymph stained with Rhodamine-labeled PNA and incubated for 8–15 minutes. (**C**) Brighter red corresponds to higher protein concentration. The labeled proteins line the outer walls of the hemocytes inside the formed aggregates. (**D**) A composite image of phase-enhanced (greyscale) and fluorescence (red) micrographs of formed hemocyte aggregates. The phase-enhanced portion of the image reveals hemocyte aggregates, and the fluorescent portion of the image reveals glycosylated proteins in the aggregates.

To clarify the physical mechanisms governing cell aggregation, time-lapse analysis was conducted. Drops of blood were placed under an inverted transmitted-light phase-enhanced microscope (Nikon Eclipse Ti; 40X Oil Objective, S Fluor, NA = 1.30, DIC H/N2; Photometrics CoolSnap HQ2 camera, 272 ms exposure, time interval = 1 sec) and cell behavior was recorded. At 10 minutes, the cells were loosely packed with an amorphous material in the intercellular spaces of the aggregates (Fig. 2A). The optical density of this amorphous material differed from that of the cells. As the clot matured (Fig. 2B), the cells packed more closely, with the amorphous material becoming significantly darker and denser (Supplementary material).

Staining the hemolymph with rhodamine-labeled peanut agglutinin (PNA), which has been used for labeling glycosylated clotting proteins of *Drosophila*^24,26,30^, *Galleria*^31^, and other species^25^, reveals (Fig. 2C,D) that the hemocyte aggregates contain glycosylated proteins holding hemocytes together. Previous phase-contrast microscopy of larval hemolymph of Lepidoptera was not able to identify these modular features of the aggregates^32^.

**Figure 3:**
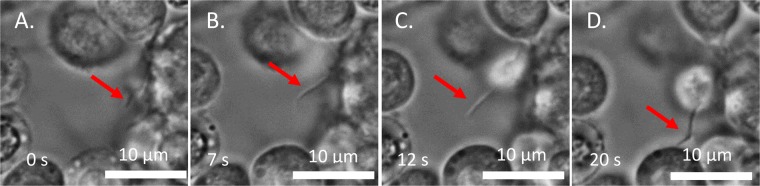
Growth of a single pseudopodial thread (red arrow). The thread bends as it extends, eventually adhering to the surface of another cell.

**Figure 4:**
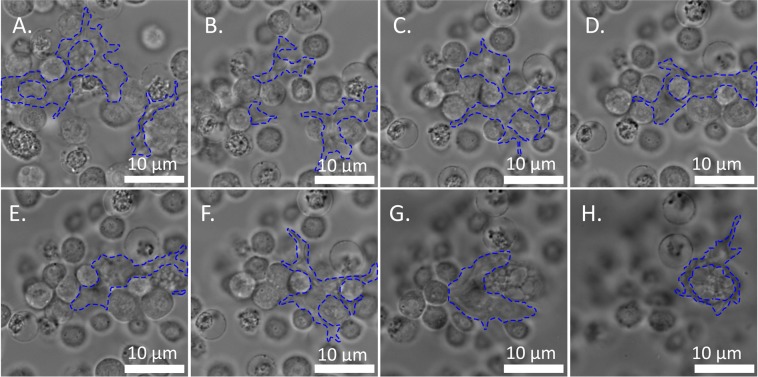
Time-lapse developmental series of pseudopodial extensions and intercellular matrix (outlined in blue). The extensions originate from only a few cells and can stretch for tens of microns. (See SI for video).

Further inspection of time-lapse videos revealed the physical mechanism of cell aggregation in the first step: it was facilitated by pseudopodia – thread-like extensions of the cells^17^ (Fig. 3). Each thread extends from the originating cell and bends on its way to another cell to which it adheres. The pseudopodia originate from only a few cells and change shape with time (Fig. 4). The thread-like pseudopodia adhere to remote cells and pull them closer to the originating cell. Thus, the cell sending out pseudopodia functions as a spring gun: the thread is presumably stressed, thus stretching the constituent material on its way to finding the target. When a cell is contacted, the elastic stress in the pseudopodium tends to relax, similar to a spring contracting back to the gun, bringing the targeted entity with it. Thus, the aggregates grow larger and change their shape. In some insects, pseudopodial networks and meshes occlude other blood cells, developing elastic, contractile blobs that are not necessarily isolated from one another^17^.

**Figure 5:**
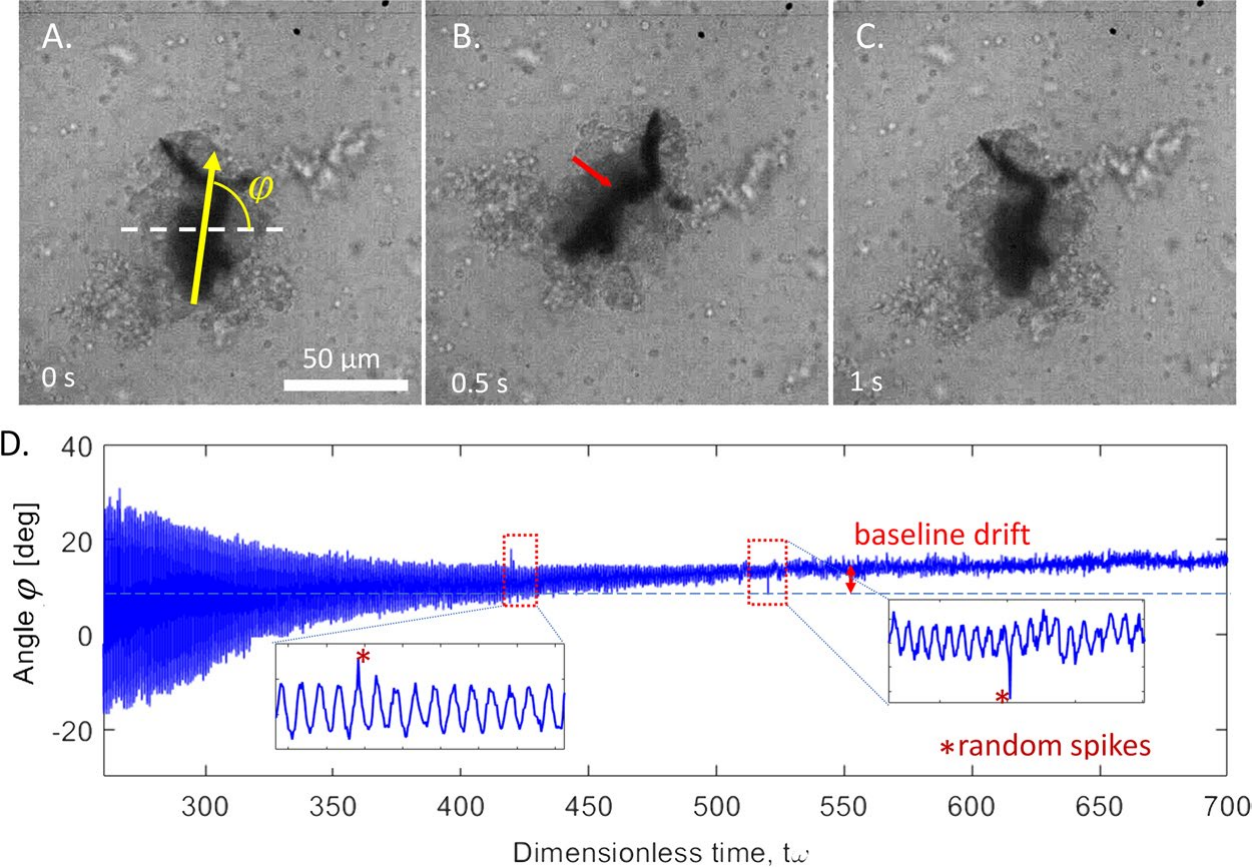
Rotation of a cluster of Ni nanorods embedded in the cell aggregate. (**A**–**C**) Snapshots of the aggregate oscillations detected five minutes after insect wounding and blood extraction. The panels show the maximum and the minimum declinations from the mean orientation during one period of oscillation. The dark cluster of Ni nanorods is indicated with a red arrow in panel B. The cells form a large grey clump around the nanorods. (**D**) Time dependence of angle *φ*. The amplitude of oscillations decreases with time exponentially, $\propto \,\exp (\,-\,{t}/{\tau })$, which signifies an increasing rigidity of the material. The specifics of the probe fluctuations are illustrated in the inserts. In this example, τ = 77 s.

To probe the kinetics of aggregate densification, we exploited the ability of foreign particles to trigger the coagulation reaction of insect blood^33–35^. Nickel nanorods with ferromagnetic properties coated with poly(vinylpyrrolidone) were dispersed in a drop of blood and forced to continuously rotate by applying a B = 1.5 mT rotating magnetic field with a *ω* = 1Hz frequency^36^. When an applied rotating magnetic field B acts on a ferromagnetic probe of magnetization M and volume V, it exerts mechanical torque, ${\tau }_{{\rm{m}}}=VMB\,\sin (\omega t-\phi )$, where t is time and *φ* is the angle that the probe makes with a stationary reference axis, the dashed line in Fig. 5. A cluster of nickel nanorods is the darkest domain and the grayish area is the cell aggregate containing hemocytes and connecting proteinaceous material (Fig. 5). Initially, the nanorods either made full revolutions or rotated with some oscillations around a mean direction of rotation, similar to how they behave in water^37,38^. This free rotation occurred for several seconds after initial dispersion of nanorods in the sample. Within three minutes, the cells began adhering to each other, anchored by pseudopodia; the pseudopodia also stuck to the nanorods, engulfing them in the cell aggregates. As the aggregates stopped growing and began shrinking, the cells packed more closely and the aggregates darkened. During aggregate densification and darkening, the amplitude of the probe oscillations decreased exponentially with time and finally approached zero when the probe became immobilized in the matrix (Fig. 5).

**Figure 6:**
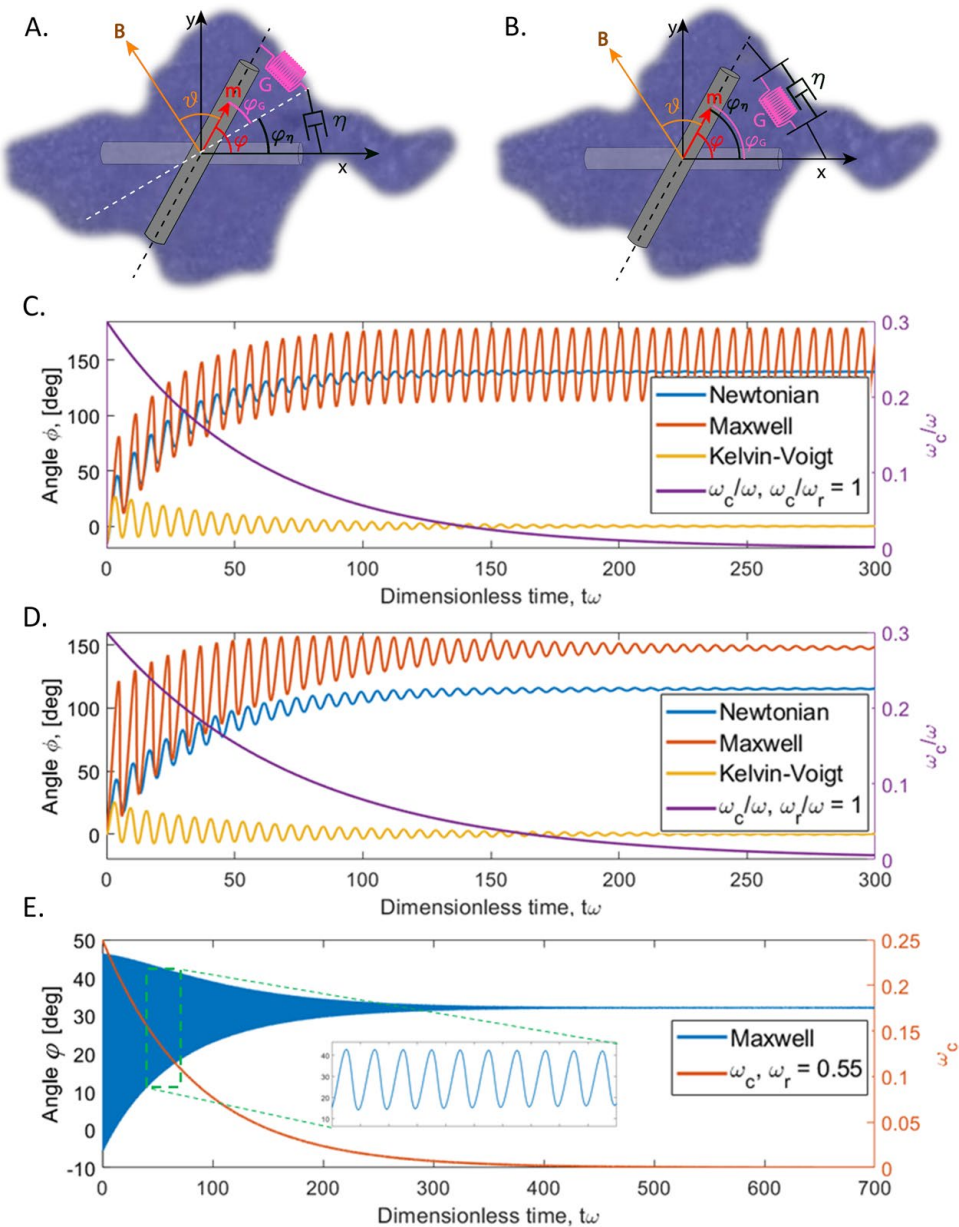
Schematics of two scenarios of material response to probe oscillation. The probe is modeled as a rod with its original horizontal position, **B** is the vector of applied magnetic field and **m** is magnetic moment of the nanorod. The probe is surrounded by a cellular matrix. A Newtonian scenario is analyzed for reference. (**A**) The probe is partially embedded in an elastic aggregate and exposed to viscous fluid. The elastic and viscous drag are added in series. (**B**) The probe is completely embedded in the gel-like aggregate, which produces an elastic and a viscous torque simultaneously; hence, the spring and dashpot are in parallel. The symbols are explained in the text. (**C**–**E**) Numerical analysis of rotation of a ferromagnetic probe in viscous Newtonian (blue), Maxwell (orange), and Kelvin-Voigt (yellow) materials. For illustration, the following parameters were used in calculations ω_c_/ω_r_ = 1, ${\omega }_{c}(t\omega )/\omega ={\tilde{\omega }}_{c0}\,\exp (\,-\,t\omega /\tau \omega )$, and ${\tilde{\omega }}_{c0}$ = 0.3, $\tau \omega \,=$ 75. (**C**) Elastic modulus is constant and viscosity increases exponentially. (**D**) Elastic modulus and viscosity increase exponentially. (**E**) A plot of the behavior of the Maxwell model using the experimentally obtained parameters: ${\tilde{\omega }}_{c0}$ = 0.25, τω = 85, ω_r_ = 0.55. The theoretically obtained profile is similar to the experimentally obtained profile in Fig. 5D.

We propose two scenarios describing exponentially fading oscillations of a magnetic probe. In the first scenario, the probe might be hinged to the cell aggregate, remaining partially exposed to the fluid, as the two-dimensional image of the cell aggregate cannot guarantee that the probe is not protruding from the aggregate in the z-direction. Thus, when the probe is forced to rotate in response to the magnetic torque, it experiences an elastic reaction from the aggregate and a viscous drag from the fluid (Fig. 6A). This scenario can be modeled with the Maxwell model, where the spring models the elastic reaction of the aggregate matrix and the dashpot (damper) models the viscous friction of the fluid^39,40^.

In the second scenario, the probe might be completely embedded in a gel-like material formed by the cells and the connecting medium (Fig. 6B). The gel-like material responds elastically to the applied oscillating magnetic torque. Upon deformation of the matrix, a gel-like aggregate squeezes out plasma. This plasma flow contributes to a viscous drag on the magnetic probe, which is modeled as a dashpot (Fig. 6B). Thus, in the second scenario, a dashpot modeling viscous friction and an elastic spring modeling elastic response of the gel, both set in parallel, oppose the magnetic torque. This second scenario corresponds to the Kelvin-Voigt model of viscoelasticity^37,38^.

In the Maxwell model (first scenario), the viscous, *τ*_*η*_, elastic, *τ*_*G*_, and magnetic, *τ*_m_, torques are equal to each other *τ*_*η*_ = *τ*_*G*_ = *τ*_m_. Introducing the drag coefficient γ, which is proportional to viscosity, *η*, and an effective shear modulus of the aggregate, *G*, the viscous and elastic torques are written as *τ*_*η*_ = *γ*d*φ*_*η*_/d*t* and *τ*_*G*_ = *γG φ*_*G*_*/η*, where *φ*_*η*_ and *φ*_*G*_ are the rotation angles associated with the spring and dashpot displacements, respectively, and *t* is time. The angular displacement of the probe satisfies the relation *φ*_*η*_ + *φ*_*G*_ = *φ*. Thus, the torque balance provides the equation governing probe rotation^39^:

1 $$\frac{{\rm{d}}\phi }{{\rm{d}}t}-\frac{\eta }{\gamma G}\frac{{\rm{d}}{\tau }_{{\rm{m}}}}{{\rm{d}}t}=\frac{{\tau }_{{\rm{m}}}}{\gamma }$$

In the Kelvin-Voigt model (second scenario), when the dashpot and the spring are in parallel, the torques satisfy the relation *τ*_m_ = *τ*_*η*_ + *τ*_*G*_, and the angular displacements are the same for the spring and dashpot, *φ*_*η*_ = *φ*_*G*_ = *φ*. The torque balance for the Kelvin-Voigt model is thus:

2 $$\frac{{\rm{d}}\phi }{{\rm{d}}t}+\frac{G}{\eta }\phi =\frac{{\tau }_{{\rm{m}}}}{\gamma }$$

When the second term in eqs (1 and 2) is absent, these two models become identical and describe the probe behavior in a Newtonian liquid, which does not sustain any shear stress and hence does not show any elastic response. While this Newtonian model clearly does not describe the material behavior observed in experiment, it still serves as a useful reference for theoretical analysis.

In experiments, the amplitude of the probe oscillations decreases exponentially with time, $\phi \,\propto \,exp(\,-\,{\rm{t}}/{\rm{\tau }})$, which signifies an internal timescale *τ* for the materials reaction, perhaps associated with the molecular immune cascade reaction. We investigated the probe reaction on these changes by modeling viscosity, *η*, as an exponentially increasing function of time, ${\rm{\eta }}\propto exp({\rm{t}}/{\rm{\tau }})$, with two alternatives: a) the elastic modulus *G* remaining constant and b) the elastic modulus increasing with time exponentially, ${\rm{G}}\propto \,exp({\rm{t}}/{\rm{\tau }})$, so that the ratio *ω*_r_ = *G*/*η* remains constant. The latter alternative is inspired by the Green-Tobolsky temporary filamentary network theory of gels, assuming that the aggregate is held intact by polymeric filaments physically restrained by temporary junctions that spontaneously form and break at the probability 1/τ per unit time^41^. Each temporary junction also contributes to the viscous friction.

The probe behavior for the viscous fluid (Newtonian model), Maxwell, and Kelvin-Voigt models is presented in Fig. 6(C–E). When viscosity increases with time while the elastic modulus remains constant, the oscillations around the mean exhibit decreasing amplitude in the Newtonian and Kelvin-Voigt models but do not change in the Maxwell model. The mean value of the rotation angle in the Newtonian and Maxwell models approaches some non-zero value while the slope of the mean decreases and approaches 0. The mean for the Kelvin-Voigt model does not change and remains at 0. When viscosity and the elastic modulus both increase with time at the same rate, the probe in a Maxwell fluid experiences decreasing amplitude of oscillation around the mean and the slope of the mean decreases and approaches 0. The behavior of the Kelvin-Voigt probe, while not quantitatively identical to the first case, does not seem to have any qualitatively distinguishable features. At low values of characteristic frequency *ω*_c/_*ω* = *MBV*/(*ωγ)*, both Maxwell and Kelvin-Voigt models show decreasing oscillations with a non-changing mean angle. In this scenario, both models represent similar materials that are elastic; it is difficult to qualitatively distinguish probe behavior in these two materials.

Summarizing the theoretical analysis (Supplementary Material), we conclude that the aggregate material represented by the Maxwell and Kelvin-Voigt models, with viscosity increasing exponentially over time, can explain experimental features of probe rotation. In the Kelvin-Voigt model, the material viscosity increases exponentially with time and the elastic modulus either increases at the same rate or remains constant. In the Maxwell model, both viscosity and the elastic modulus of the material increase exponentially with time. This analysis points to a dominant role for exponentially increasing viscosity as the main factor hindering probe rotation. It favors the proposed scenario that the effect of blood thickening mostly comes from macromolecules, presumably produced during the immune cascade reactions.

The experimental challenge preventing us from evaluating the aggregate viscosity and elastic modulus for analyzing probe trajectories is aggregation of nanorods into a complexly shaped cluster. Available characterization tools do not allow estimation of its volume or magnetic moment. We can, however, evaluate the amplitude of probe oscillations, ${\xi }_{A}$, extracting it from the raw data (Supplementary material). The extracted envelope of the data sets was successfully fitted with an exponential function ${\xi }_{A}={\xi }_{A0}\exp (-t/\tau )$ with a constant prefactor ${\xi }_{A0}$(Fig. S1), where the characteristic time of the cell aggregate rigidification was estimated as τ = 77.1 s. Time-evolution of three samples from three larvae was successfully analyzed in this fashion to obtain the characteristic time of cell aggregate rigidification as τ = 86 ± 17 s. After this time, at the second step of cell aggregation, the formed clot is highly viscous and able to significantly reduce fluid mobility, preventing its flow to the wound surface and, hence, evaporation. It is rigid and able to stem bleeding and bacterial invasion.

The large surface to volume ratio of arthropods carries the problem of water loss, which is exacerbated when the cuticle is breached. Closure of wounds should be under strong selection pressure to rapidly stem water and solute loss, maintain hydrostatic pressure, and form a barrier to infection. Our quantitative analysis of cell aggregation and aggregate rigidification revealed that the limiting step in clot formation is cell aggregation. In our experiments at 20–22 °C, the blood cells formed soft aggregates in about 3 minutes. Aggregate rigidification and densification then happened faster, within ~1.2 minutes. The total time for formation of a rigid cell aggregate is, therefore, ~4 minutes. This time is incomparably shorter than that for the formation of a scab and new epidermal tissue by more than two orders of magnitude^1^ The initial clot not only rapidly stanches fluid loss, but also may provide scaffolding for the slower process of epithelial and cuticular repair^5^. The period from initiation of the molecular cascade to the first microscopically visual detection of cell aggregation remains the unquantified bridge between molecular biology and materials science. Many studies and protocols for hemolymph clotting use red blood cells for initiation of the immune cascade reaction in insects^24^. Combined with biochemical and biomolecular studies, our findings can be used to design fast-working thickeners for vertebrate blood including human blood.

The similar, but qualitative, observations of clotting across a diverse range of insect taxa^14^ suggests that our quantitative, two-step *Manduca* model describing physical features of blood-cell aggregation has general applicability. We caution, however, that the same properties that make *M*. *sexta* an attractive model—large size and ease of obtaining large volumes of hemocyte-rich blood—also raise questions about the general applicability of our findings. For example, *M*. *sexta* is highly resistant to pathogenic bacteria, compared with other lepidopterans, presumably because of increased levels of hemocytes in the blood^42^. To test the applicability of our model, we suggest that additional species of insects, with attention to gender^43^, be subjected to comparative investigation using our analytical template. We, nonetheless, expect that the general mechanism underlying the physical features for rapid wound repair has an ancient evolutionary signature, in much the same way that innate immunity reflects an origin deep in the common ancestry of invertebrates and vertebrates^44^. Cell aggregation, for instance, relies on cell mobility, inherent in blood cells across vertebrates and invertebrates, which can influence the arrangement and packing of the surrounding area^45,46^.

## Materials and Methods

### Nanorod synthesis and preparation

Nickel nanorods were prepared via electrochemical deposition into the 200-nm pores of aluminum-oxide membranes (Whatman, type). After deposition, the membrane was dissolved in an acid and the nanorods were released. (The protocol for the nanorod synthesis is described in detail in^47^) The rods were then drop-casted into a 20 mg/ml aqueous solution of molecular weight of 360000 polyvinylpyrrolidone (PVP) and left overnight in a sonicator bath. The nanorods were magnetically extracted from the PVP solution, washed in DI water, dried under dry nitrogen, and transferred into pure methanol and stored.

### Dispersion of nanorods in the sample

Nickel nanorods, 200 nm in diameter and roughly 10 μm in length were deposited in the larval blood samples. First, the nanorods suspended in methanol were deposited on a glass slide. The methanol was allowed to evaporate, leaving the nanorods on the slide. An incision was then made on the 3^rd^ proleg of the larva and several droplets of blood were dripped onto the slide. No additional interaction with the sample was performed to preserve any structure present in hemolymph. The sample was then transferred to an environmental chamber with nitrogen, and with 100% humidity, inside the magnetic rotator; the rotating magnetic field was turned on and the sample was imaged under bright-field microscopy.

### Larval maintenance

Larvae of *M*. *sexta* were obtained from Carolina Biological Supply (https://www.carolina.com) or reared in-house on diet from Great Lakes Hornworms (https://www.greatlakeshornworm.com/), with a few larvae feeding *ad libitum* on hornworm diet from Carolina Biological Supply. Deposited eggs hatched in an enclosure (humidity ca. 65% and temperature ca. 27 °C). First instars were moved to wide-mouth liter glass jars with strips (ca. 3 × 15 cm) of plastic gutter guard (Frost King Model VX620) inside as a climbing substrate and food support. Larvae were held at room temperature (about 25 °C) and 24 h artificial light. To provide gas exchange and reduce humidity, aluminum window screening was cut to fit the jar bands. Diet (ca. 10 ml) was added for the first three instars as needed. For later instars, larvae were removed from jars and placed in clean jars with diet added as it was consumed (at least twice a week). The number of larvae per jar was reduced over time, with 10 or fewer last instars per jar. Larvae that were moving to the prepupation stage were not used for hemolymph, as its composition changes rapidly during this stage. Prepupation larvae had a more yellowish thorax, stopped eating, and began wandering.

### Hemolymph extraction

Larvae of *M*. *sexta*, 1–2 days before pre-pupation and weighing more than 8.5 g, were washed with DI water and dried with paper towels. To prevent movement, larvae were placed in specially designed containers that restrained them along the length of the body while leaving the second and third prolegs exposed. Once the larva was secured, an incision was made with a razor blade on the third proleg. Blood exiting the wound freely was collected on a glass slide. Blood was extracted only once from each specimen. All experiments were conducted at temperatures between 20 and 22 °C.

### Hemolymph observation

Cellular aggregation during the first 10 minutes of coagulation was observed under high magnification. Drops of blood were placed under an inverted transmitted-light phase-enhanced microscope (Nikon Eclipse Ti; 40X Oil Objective, S Fluor, NA = 1.30, DIC H/N2; Photometrics CoolSnap HQ2 camera, 272 ms exposure, time interval = 1 sec) and cell behavior was recorded.

Nanorod rotation and oscillation were observed under a bright-field microscope (Olympus BX-51, Basler acA2040-180km Camera, 10x Objective), with a frame rate of 20 frames per second.

### Peanut agglutinin (PNA) labeled fluorescent imaging

Fluorescence imaging was performed using an inverted microscope (Nikon Eclipse Ti) with 20x and 60x objectives and a DAPI cube. Phase-enhanced microscopy was performed using the same set-up without the DAPI cube. Images were recorded with a black and white camera (Photometrics CoolSnap HQ2). For all images, the exposure was set to auto and the contrast look-up tables were adjusted manually for best visibility.

## Supplementary information

Clot Nucleation in Insects SI PDF

SI video
