# Supplementary material for: Nucleation and Formation of a Primary Clot in Insect Blood
Source: Sci Rep. 2019 Mar 5;9:3451. doi: 10.1038/s41598-019-40129-0 (PMC6401176; doi:10.1038/s41598-019-40129-0)
Supplement: Supplementary file 1 — Clot Nucleation in Insects SI PDF [file 41598_2019_40129_MOESM1_ESM.pdf]

Supplementary Material

Nucleation and Formation of a Primary Clot in Insect Blood

Pavel Aprelev[1], Terri F. Bruce[2], Charles E. Beard[3], Peter H. Adler[3], and Konstantin G. Kornev*[1]

[1]Department of Materials Science and Engineering, Clemson University, Clemson, South Carolina, USA, 29634. E-mail: kkornev@clemson.edu
[2]Light Imaging Facility, Clemson University, Clemson, South Carolina, USA, 29634
[3]Department of Plant and Environmental Sciences, Clemson University, Clemson, South Carolina USA, 29634

Governing equations.

When a ferromagnetic probe of volume V is subjected to an applied magnetic field B, the magnetic torque is written as $\tau_{\mathrm{m}} = VMB\sin\theta$, where M is the magnetization of the probe material and θ is the angle formed by the magnetization vector and the field vector. Substituting the magnetic torque into eqs. (1) and (2), one obtains the basic equations (38):

$$\frac{\mathrm{d}\varphi}{\mathrm{d}t} - \frac{\omega_{\mathrm{c}}}{\omega_{\mathrm{r}}}\cos\theta\frac{\mathrm{d}\theta}{\mathrm{d}t} = \omega_{\mathrm{c}}\sin\theta \quad \text{(Maxwell Model)} \qquad \text{(S.1)}$$

$$\frac{\mathrm{d}\varphi}{\mathrm{d}t} + \omega_{\mathrm{r}}\varphi = \omega_{\mathrm{c}}\sin\theta \quad \text{(Kelvin-Voigt Model)} \qquad \text{(S.2)}$$

where $\omega_{\mathrm{c}}=MBV/\gamma$, and $\omega_{\mathrm{r}}=G/\eta$ is the reciprocal to the viscoelastic relaxation time. Probe rotation in a Newtonian fluid is described by an equation without the second terms in eqs. (S1)-(S2). For an applied field rotating with frequency ω, one writes $\theta = \omega t - \varphi$. Therefore, we have

Newtonian medium:

$$\frac{d\varphi}{dt} = \omega_c\sin(\omega t - \varphi), \text{ or, in dimensionless variables:}$$
$$\frac{d\varphi}{d\tilde{t}} = \tilde{\omega}_c\sin(\tilde{t} - \varphi). \qquad \text{(S.3)}$$

Maxwell medium:

$$\frac{d\varphi}{dt} = \frac{\omega_{\mathrm{c}}\sin(\omega t - \varphi) + \omega\frac{\omega_{\mathrm{c}}}{\omega_{\mathrm{r}}}\cos(\omega t - \varphi)}{1 + \frac{\omega_{\mathrm{c}}}{\omega_{\mathrm{r}}}\cos(\omega t - \varphi)}, \text{ or, in dimensionless variables:}$$
$$\frac{d\varphi}{d\tilde{t}} = \frac{\tilde{\omega}_c\sin(\tilde{t} - \varphi) + \frac{\tilde{\omega}_{\mathrm{c}}}{\tilde{\omega}_{\mathrm{r}}}\cos(\tilde{t} - \varphi)}{1 + \frac{\tilde{\omega}_{\mathrm{c}}}{\tilde{\omega}_{\mathrm{r}}}\cos(\tilde{t} - \varphi)}.$$

(S.4)

Kelvin-Voigt medium:

$$\frac{d\varphi}{dt} = \omega_{\mathrm{c}}\sin(\omega t - \varphi) - \omega_{\mathrm{r}}\varphi, \text{ or, in dimensionless variables:}$$
$$\frac{d\varphi}{d\tilde{t}} = \tilde{\omega}_c\sin(\tilde{t} - \varphi) - \tilde{\omega}_{\mathrm{r}}\varphi, \qquad \text{(S.5)}$$

where the dimensionless parameters are introduced as follows:

$$\tilde{\omega}_c = \omega_c/\omega, \ \tilde{\omega}_r = \omega_r/\omega, \ \tilde{t} = \omega t. \qquad \text{(S.6)}$$

$\omega_c \propto \eta^{-1}$, η is viscosity, $\omega_r = \frac{G}{\eta}$, G is the elastic modulus.

In our experiments, we observed that the cell aggregates matured with time and their properties changed sufficiently fast. We will consider two cases that can be written in normalized form as $\tilde{\tau} = \omega\tau$,

$$\tilde{\omega}_c\left(\tilde{t}\right) = \tilde{\omega}_{c0}\exp\left(-\tilde{t}/\tilde{\tau}\right);\ \tilde{\omega}_c/\tilde{\omega}_r \propto G \approx const \quad \text{(S.7)}$$

$$\tilde{\omega}_c\left(\tilde{t}\right) = \tilde{\omega}_{c0}\exp\left(-\tilde{t}/\tilde{\tau}\right);\ \tilde{\omega}_r = const\text{ , i.e. } G \propto \exp\left(\tilde{t}/\tilde{\tau}\right) \quad \text{(S.8)}$$

In equation (S7), the critical frequency ω_c exponentially decreases, which corresponds to the viscosity η exponentially increasing, and ω_c/ω_r remains constant, which corresponds to the elastic modulus G remaining constant. In the case of equation (S8), ω_c again exponentially decreases, which corresponds to the viscosity exponentially increasing, and ω_r remains constant, which corresponds to the elastic modulus G exponentially increasing. The results of numeric calculations of these equations are shown in Figure 6. In both cases, we set the numerical parameters such that the characteristic time of the materials parameters change is much smaller than the period of oscillations of the probe, i.e. $\tau\omega = 75$.

It remains unknown, however, whether the two seemingly different equations describe a non-flowing, rigidifying material similarly. To answer this question, we first linearize the equations to find the dependency of the amplitude of the angular oscillations on the changing ω_c and ω_r.

Linearization of the Maxwell model at high viscosity.

Beginning with normalized equation (S.4), and seeing from experiment that as time increases indefinitely, $\tilde{t} \to \infty$ angle φ of the probe approaches a constant value, $\varphi \to \varphi_0$. Thus, at large times, we can write $\varphi = \varphi_0 - \xi$, where $|\xi| << 1$ is the amplitude of an oscillation. From experiment, we can set the value of $\varphi_0 = 0$. The trigonometric function components from these equations can thus be rewritten as:

$$\sin\left(\tilde{t} - \varphi\right) = \sin\left(\tilde{t} + \xi\right) \quad \text{(S.9)}$$

$$\cos\left(\tilde{t} - \varphi\right) = \cos\left(\tilde{t} + \xi\right) \quad \text{(S.10)}$$

Applying a trigonometric identity and Taylor expansion over ξ equation (S.9) takes the form of

$$\sin\left(\tilde{t} - \varphi\right) \approx \sin\left(\tilde{t}\right) + \xi\cos\left(\tilde{t}\right) \quad \text{(S.11)}$$

Similarly, equation (S.10) takes the form of

$$\cos\left(\tilde{t} - \varphi\right) \approx \cos\left(\tilde{t}\right) + \xi\sin\left(\tilde{t}\right) + O\left(\xi^2\right) \quad \text{(S.12)}$$

Substituting equations (S.11) and (S.12) into Maxwell equation (S.4) and factoring out ξ, we obtain

$$-\frac{d\xi}{d\tilde{t}}=\xi\left[\frac{\tilde{\omega}_c\left(\tilde{t}\right)}{\tilde{\omega}_r\left(\tilde{t}\right)}\sin\left(\tilde{t}\right)-\tilde{\omega}_c\left(\tilde{t}\right)\cos\left(\tilde{t}\right)\right]- \\ -\tilde{\omega}_c\left(\tilde{t}\right)\sin\left(\tilde{t}\right)-\frac{\tilde{\omega}_c\left(\tilde{t}\right)}{\tilde{\omega}_r\left(\tilde{t}\right)}\cos\left(\tilde{t}\right) \tag{S.13}$$

At large time $\tilde{t}$, the amplitude ξ is small and the viscosity is high (ω_c is low); thus, both terms inside the brackets are small, leaving the declination from the mean dependence as

$$\frac{d\xi}{d\tilde{t}}=\tilde{\omega}_c\left(\tilde{t}\right)\sin\left(\tilde{t}\right)+\frac{\tilde{\omega}_c\left(\tilde{t}\right)}{\tilde{\omega}_r\left(\tilde{t}\right)}\cos\left(\tilde{t}\right) \tag{S.14}$$

Now, considering only the case of exponentially increasing viscosity and constant elastic modulus (equation (S.7)), the behavior of the oscillations takes the form of

$$\frac{d\xi}{d\tilde{t}}=\tilde{\omega}_{c0}\exp\left(-\tilde{t}/\tilde{\tau}\right)\sin\left(\tilde{t}\right)+C\cos\left(\tilde{t}\right) \tag{S.15}$$

where C is a constant. This relation can now be integrated to find the dependency of the angle of the probe as a function of time for this scenario.

$$\xi=\frac{-\tilde{\omega}_{c0}\tilde{\tau}\exp\left(-\tilde{t}/\tilde{\tau}\right)}{1+\tilde{\tau}^2}\left(\tilde{\tau}\cos\left(T\right)+\sin\left(T\right)\right)+C\sin\left(T\right) \tag{S.16}$$

During the experiments, we track the amplitude of these oscillation ξ_A, and are, therefore, interested in the prefactors of the periodic function and not the phase. Thus, we aim to obtain the form of the expression, where the amplitude is easily observable. Because

$$A\sin\left(x\right)+B\cos\left(x\right)=\sqrt{A^2+B^2}\sin\left(x+\alpha\right) \tag{S.17}$$

where $\alpha=\arctan\left(B/A\right)$, equation (S.16) can be rewritten as

$$\xi=\frac{-\tilde{\omega}_{c0}\tilde{\tau}\exp\left(-\tilde{t}/\tilde{\tau}\right)}{\sqrt{\tilde{\tau}^2+1}}\sin\left(\tilde{t}+\tan^{-1}\left(\tilde{\tau}\right)\right)+C\sin\left(\tilde{t}\right) \tag{S.18}$$

Furthermore, the amplitude of the sum of amplitudes of two sine functions with an arbitrary phase shift, $A\sin\left(x+\alpha\right)+B\sin\left(x\right)$, can be shown to equal $\sqrt{A^2+B^2+2AB\cos\left(a\right)}$. Thus, the amplitude of the oscillations for the current case is expressed as

$$\xi_A=\sqrt{\left(\frac{\tilde{\omega}_{c0}\tilde{\tau}\exp\left(-\tilde{t}/\tilde{\tau}\right)}{\sqrt{\tilde{\tau}^2+1}}\right)^2+C^2-\frac{2C\tilde{\omega}_{c0}\tilde{\tau}\exp\left(-\tilde{t}/\tilde{\tau}\right)}{\tilde{\tau}^2+1}} \tag{S.19}$$

In the experiments, $\tilde{\tau}>>1$; thus, the expression can be Taylor expanded to

$$\xi_A = \sqrt{\tilde{\omega}_{c0}{}^2 \exp\left(-2\tilde{t}/\tilde{\tau}\right) + C^2 - \frac{2C\tilde{\omega}_{c0}}{\tilde{\tau}} \exp\left(-\tilde{t}/\tilde{\omega}\right) + O\left(\frac{1}{\tilde{\tau}^2}\right)} \tag{S.20}$$

The kinetics of the amplitude of oscillation of a probe rotating in a Maxwell liquid with properties changing according to equation (S.7) develop as follows. Initially, when $\tilde{t}$ is small, depending on the elastic modulus and thus C, $\tilde{\omega}_{c0}$ contributes a significant portion to the amplitude. As time goes on, the contribution of $\tilde{\omega}_{c0}$ becomes less significant and the amplitude approaches C. This is consistent with the behavior of the Maxwell model in Figure 6 C and does not represent the experimental data. Thus, this case will no longer be considered.

We now consider the behavior of amplitude in the case of exponentially increasing viscosity and exponentially increasing elastic modulus (equation(S.8)). Starting with equation (S.14), we substitute the explicit relations of $\tilde{\omega}_c$ and $\tilde{\omega}_r$ in time,

$$\frac{d\xi}{d\tilde{t}} = \tilde{\omega}_{c0} \exp\left(-\tilde{t}/\tilde{\tau}\right)\sin\left(\tilde{t}\right) + \frac{\tilde{\omega}_{c0}}{\tilde{\omega}_r} \exp\left(-\tilde{t}/\tilde{\tau}\right)\cos\left(\tilde{t}\right) \tag{S.21}$$

Again, we integrate to find the dependency of the angle of the probe as a function of time for this scenario.

$$\xi = -\frac{\tilde{\omega}_{c0}\tilde{\tau}\exp\left(-\tilde{t}/\tilde{\tau}\right)}{1+\tilde{\tau}^2}\left(\tilde{\tau}\cos\left(\tilde{t}\right) + \sin\left(\tilde{t}\right)\right) + \frac{\tilde{\omega}_{c0}\tilde{\tau}\exp\left(-\tilde{t}/\tilde{\tau}\right)}{\tilde{\omega}_r\left(1+\tilde{\tau}^2\right)}\left(-\cos\left(\tilde{t}\right) + \tilde{\tau}\sin\left(\tilde{t}\right)\right) \tag{S.22}$$

Applying equation (S.17),

$$\xi = \frac{-\tilde{\omega}_{c0}\tilde{\tau}\exp\left(-\tilde{t}/\tilde{\tau}\right)}{\sqrt{\tilde{\tau}^2+1}}\sin\left(\tilde{t} + \tan^{-1}\left(\tilde{\tau}\right)\right) + \frac{\tilde{\omega}_{c0}\tilde{\tau}\exp\left(-\tilde{t}/\tilde{\tau}\right)}{\tilde{\omega}_r\sqrt{\tilde{\tau}^2+1}}\left(\sin\left(\tilde{t} + \tan^{-1}\left(-1/\tilde{\tau}\right)\right)\right) \tag{S.23}$$

Writing the dependence of the amplitude as a function of time, we get

$$\xi_A = \sqrt{f_1 + f_2}$$
$$f_1 = \left(\frac{-\tilde{\omega}_{c0}\tilde{\tau}\exp\left(-\tilde{t}/\tilde{\tau}\right)}{\tilde{\tau}^2+1} + \frac{\tilde{\omega}_{c0}\tilde{\tau}\exp\left(-\tilde{t}/\tilde{\tau}\right)}{\tilde{\omega}_r\sqrt{\tilde{\tau}^2+1}\sqrt{\tilde{\tau}^{-2}+1}}\right)^2 \tag{S.24}$$
$$f_2 = \left(\frac{-\tilde{\omega}_{c0}\tilde{\tau}^2\exp\left(-\tilde{t}/\tilde{\tau}\right)}{\tilde{\tau}^2+1} - \frac{\tilde{\omega}_{c0}\tilde{\tau}\exp\left(-\tilde{t}/\tilde{\tau}\right)}{\tilde{\tau}\tilde{\omega}_r\sqrt{\tilde{\tau}^2+1}\sqrt{\tilde{\tau}^{-2}+1}}\right)^2$$

In experiment $\tilde{\tau} >> 1$; thus, the expression can be simplified to

$$f_1 = \left(\frac{-\tilde{\omega}_{c0} \exp\left(-\tilde{t}/\tilde{\tau}\right)}{\tilde{\tau}} + \frac{\tilde{\omega}_{c0} \exp\left(-\tilde{t}/\tilde{\tau}\right)}{\tilde{\tau}\tilde{\omega}_r} + O\left(\frac{1}{\tilde{\tau}^2}\right) \right)^2$$
$$f_2 = \left(-\tilde{\omega}_{c0} \exp\left(-\tilde{t}/\tilde{\tau}\right) - \frac{\tilde{\omega}_{c0} \exp\left(-\tilde{t}/\tilde{\tau}\right)}{\tilde{\tau}\tilde{\omega}_r} + O\left(\frac{1}{\tilde{\tau}^2}\right) \right)^2 \tag{S.25}$$

and algebraically expanded to

$$f_1 = \frac{\tilde{\omega}_{c0}{}^2 \exp\left(-2\tilde{t}/\tilde{\tau}\right)}{\tilde{\tau}^2} + \frac{\tilde{\omega}_{c0}{}^2 \exp\left(-2\tilde{t}/\tilde{\tau}\right)}{\tilde{\tau}^2\tilde{\omega}_r{}^2} - 2\frac{\tilde{\omega}_{c0}{}^2 \exp\left(-2\tilde{t}/\tilde{\tau}\right)}{\tau^2\omega^3\omega_r}$$
$$f_2 = \frac{\omega_{c0}{}^2 \exp\left(-2\tilde{t}/\omega\tau\right)}{\omega^2} + \frac{\omega_{c0}{}^2 \exp\left(-2\tilde{t}/\omega\tau\right)}{\tau^2\omega^2\omega_r{}^2} + 2 \tag{S.26}$$

The exponent and $\tilde{\omega}_{c0}{}^2$ terms can be factored out to

$$\xi_A = \tilde{\omega}_{c0} \exp\left(-\tilde{t}/\tilde{\tau}\right)\sqrt{1 + \frac{2}{\tilde{\tau}\tilde{\omega}_r} + \frac{1}{\tilde{\tau}^2} + \frac{2}{\tilde{\tau}^2\tilde{\omega}_r{}^2} - \frac{2}{\tilde{\tau}^2\tilde{\omega}_r}} \tag{S.27}$$

Linearization of the Kelvin-Voigt model

Similar steps as above will be performed for the Kelvin-Voigt model for material changing according to equations (S.7) and (S.8). Beginning with the normalized equation(S.5), we write the expression for deviations of the angle φ from the mean ξ at large $\tilde{t}$ as

$$\frac{d\xi}{d\tilde{t}} = -\xi\left[\tilde{\omega}_{\mathrm{r}}\left(\tilde{t}\right) + \tilde{\omega}_c\left(\tilde{t}\right)\cos\left(\tilde{t}\right)\right] - \tilde{\omega}_c\left(\tilde{t}\right)\sin\left(\tilde{t}\right) \tag{S.28}$$

At large time $\tilde{t}$, amplitude ξ is small and the viscosity is high ($\tilde{\omega}_c$ is low); thus, the second bracketed term can be neglected. Thus,

$$\frac{d\xi}{d\tilde{t}} = -\xi\tilde{\omega}_{\mathrm{r}}\left(\tilde{t}\right) - \tilde{\omega}_c\left(\tilde{t}\right)\sin\left(\tilde{t}\right) \tag{S.29}$$

Now, we consider only the case of exponentially increasing viscosity and constant elastic modulus (equation (S.7)). At large time $\tilde{t}$, the amplitude ξ and $\tilde{\omega}_r$ are small. The first term can thus be ignored. Plugging the relation from equation (S.7) into (S.29), the profile of the oscillations takes the form of

$$\frac{d\xi}{d\tilde{t}} = -\tilde{\omega}_{c0} \exp\left(-\tilde{t}/\tilde{\tau}\right)\sin\left(\tilde{t}\right) \tag{S.30}$$

This relation can now be integrated to find the dependency of the angle of the probe in a Kelvin-Voigt medium as a function of time for this scenario.

$$\xi = \frac{-\tilde{\omega}_{c0}\tilde{\tau}\exp\left(-\tilde{t}/\tilde{\tau}\right)}{1+\tilde{\tau}^2}\left(\tilde{\tau}\cos\left(\tilde{t}\right)+\sin\left(\tilde{t}\right)\right) \tag{S.31}$$

Using equation (S.17), we can write the behavior of the amplitude as

$$\xi_A = \frac{\tilde{\omega}_{c0}\tilde{\tau}\exp\left(-\tilde{t}/\tilde{\tau}\right)}{\sqrt{\tilde{\tau}^2+1}} \tag{S.32}$$

In the experiments $\tilde{\tau} >> 1$; thus, the expression can be simplified to

$$\xi_A = \tilde{\omega}_{c0}\exp\left(-\tilde{t}/\tilde{\tau}\right)+O\left(\frac{1}{\tilde{\tau}}\right) \tag{S.33}$$

Performing a similar analysis for the second case, where viscosity and the elastic modulus increase exponentially, and starting with equation (S.29) and plugging equations (S.8) for $\tilde{\omega}_c$ and $\tilde{\omega}_r$,

$$\frac{d\xi}{d\tilde{t}} = -\xi\tilde{\omega}_{\mathrm{r}} - \tilde{\omega}_{c0}\exp\left(-\tilde{t}/\tilde{\tau}\right)\sin\left(\tilde{t}\right) \tag{S.34}$$

Integrating, we get

$$\xi = \tilde{\omega}_{c0}\exp\left(-\tilde{t}/\tilde{\tau}\right)\cos\left(\tilde{t}\right)-\exp\left(-\tilde{t}\tilde{\omega}_r\right) \tag{S.35}$$

Again, keeping only the amplitude of the oscillating component, we have

$$\xi_A = \tilde{\omega}_{c0}\exp\left(-\tilde{t}/\tilde{\tau}\right)-\exp\left(-\tilde{t}\tilde{\omega}_r\right) \tag{S.36}$$

When $\tilde{\omega}_r > 1/\tilde{\tau}$, the positive left term decreases more slowly than the negative left term.

Equations (S.27), (S.33), and (S.36) that describe the amplitude of the oscillations of the probe in the Maxwell and Kelvin-Voigt liquids with exponentially increasing viscosity and elastic modulus, as well as the Kelvin Voigt liquid exponentially increasing viscosity and non-changing elastic modulus, exhibit some similarities. Namely, all contain the exponentially decaying term $\tilde{\omega}_{c0}\exp\left(-\tilde{t}/\tilde{\tau}\right)$. Equation (S.27) contains terms where $1/\tilde{\tau}$ competes with $1/\tilde{\omega}_r$. From experiment, we see that the time it takes the amplitudes to decay is large, $\tilde{\tau} >> 1$ and $1/\tilde{\tau} << 1$. For the $1/\tilde{\tau}\tilde{\omega}_r$ terms to be significant, $\tilde{\omega}_r$ needs to be small, which would constitute a soft material – a property we do not expect from a blood clot. Similarly, equation (S.36) contains a positive exponential with the decay rate $1/\tilde{\tau}$ and a negative exponential with a decay rate $\tilde{\omega}_r$. When $\tilde{\omega}_r > 1/\tilde{\tau}$, the positive

left term decreases more slowly than the negative left term and dictates the kinetics of the equation. We would expect this from a relatively elastic material, presumably like a clot. Thus, the driving term in all three cases is $\tilde{\omega}_{c0} \exp\left(-\tilde{t}/\tilde{\tau}\right)$ and the amplitude in all three cases is described by

$$\xi_A = \tilde{\omega}_{c0} \exp\left(-\tilde{t}/\tilde{\tau}\right) \tag{S.37}$$

In the context of this experiment, the three cases are thus indistinguishable from each other and are equivalent in their description of the physical phenomenon studied.

Analysis of probe motion

The analysis of the probe motion in cell-rich blood has experimental challenges that prevent us from analyzing the rotating trajectories of individual nanorod probes. In inert or slowly changing samples, the probe aggregates can be broken manually with a large glass rod shortly after their dispersion in the sample. In cell-rich blood, however, the cells begin adhering to the surfaces and forming the structures almost immediately, rendering any manual manipulation of the sample destructive. The probes thus have a complex geometric shape, as they consist of nanorod agglomerates. We are thus unable to determine the magnetic torque applied on the probes, and as a result, we calculate the absolute viscosity and elastic modulus of the material. Thus, only the relative properties of the cell aggregate can be determined versus time.

Another experimental challenge is that the probes are obstructed by hemocyte aggregates. This makes the algorithm for extraction of the orientation of the probe less precise and prevents us from looking at the fine features of probe behavior. We thus resolve to track only the amplitude ξ_A of these oscillations to infer the changing material properties. ξ_A is extracted from the raw data in two steps. First, the data are filtered with a frequency filter. And second, the envelope of the oscillating function is numerically extracted.

The frequency filter is first applied to remove slow drift in the average orientation of the aggregate and sharp noise spikes that occur during image analysis. The apparent drift in the mean orientation, which can be seen from panel G in Fig. 1, is caused by the structural change of the aggregate, which does not reflect the response of the material to our manipulation. The random spikes in the extracted data, which can be seen in the inserts of panel G, are created during the video analysis and also do not represent the response of the material to external perturbation. Both types of signal defects can be corrected for by applying a frequency filter on the data.

A frequency filter is a method of data manipulation that removes undesired frequencies from the signal. In our case, the oscillations of the probe due to the magnetic field have a frequency of 1 Hz. Relative to these oscillations, the drift in the baseline is a low-frequency process and the noise is a high-frequency defect. By applying a band-pass filter, we can eliminate all undesired effects. The filter was created using the MATLAB filter designer to create a narrow band filter that removes any oscillating components of the signal that are not within the 0.5 – 1.5 Hz region. The specific parameters of the filter are presented in the methods section. Once the data are passed through the filter, only the relevant data of the motion due to the magnetic perturbation remains (Supplementary Figure S1, green) and ξ_A can be extracted.

The amplitude of the oscillations ξ_A as a function of time was obtained by extracting the envelope of the filtered data (Supplementary Figure S1, blue). An envelope of an oscillating function is a curve tracing its maxima. The envelope was obtained using the MATLAB *envelope()*

function, which calculates the upper and lower envelopes, using a discrete Fourier transform of the data. The function creates a smooth curve, connecting the maxima. The insert in Supplementary Figure S1 shows a close-up of the resultant envelope.

The extracted envelope of the data was then fitted with an exponential function, $\xi_A = \xi_{A0} \exp\left(-\tilde{t} / \tilde{\tau}\right)$. An example of the fit of a dataset from a single sample with decay time of $\tilde{\tau}$ = 77.1s is presented in Supplementary Figure S1. Time-evolution of three samples from three caterpillars was successfully analyzed in this fashion to obtain the value of $\tilde{\tau}$ = 86 ±17. Because $\tilde{\tau} = \omega\tau$ and in the experiment $\omega = 1$ Hz, the characteristic time of the soft clot exponential rigidification is τ = 86 ± 17s.

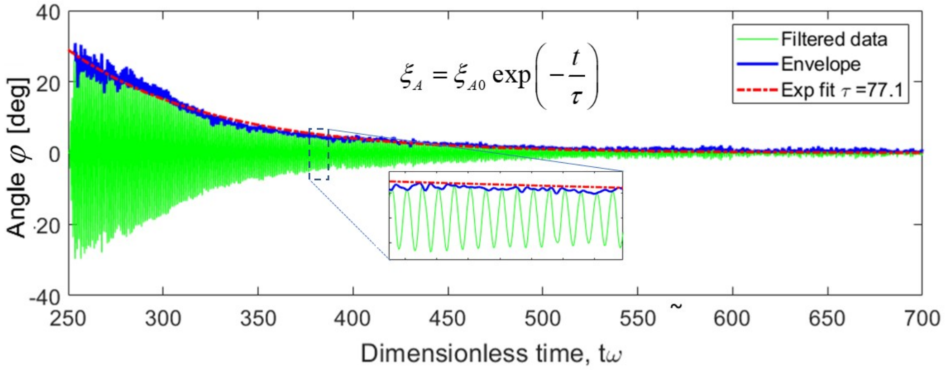

Supplementary Figure S1. The (green) filtered oscillation of the magnetic probe embedded in an aggregate of blood cells during clotting in response to the applied rotating magnetic field. The data have been (blue) enveloped, and (red dashed) fitted with an exponential function.

Fit of a Maxwell model during initial oscillations

Several initial oscillations of the probe were used to estimate the critical frequencies ω_c and ω_r using the Maxwell model, under the assumption that these properties do not change during the short time-window of the measurement. The data were fitted using equation (1) with ω_c and ω_r as fitting parameters. The results are presented in Supplementary Figure S2.

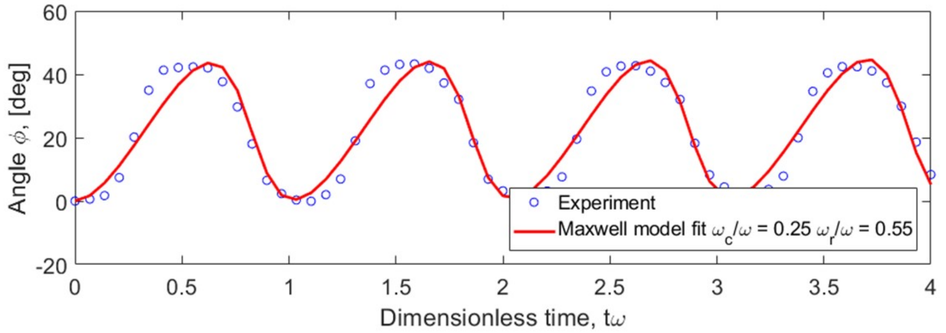

Supplementary Figure S2. An example fit of the experimental data with a Maxwell model. The viscosity and the elastic modulus of the material during the presented time period are assumed to be constant. The best fit of the data resulted in ω_c/ω= 0.25 and ω_r/ω= 0.55,

Filtering of data

The filter was implemented using the MATLAB *filter()* function, for which a custom filter was created using the MATLAB Filter Designer. The designed filter was a narrow band band-pass filter that removes any undesired oscillations not within the 0.5 – 1.5 Hz region. We chose a

Butterworth filter as it has a maximally flat frequency response and does not disrupt the useful signal. The parameters specified for the function are presented in Table S1 and the frequency response of the filter is presented in Supplementary Figure S3. The band width of the filter was chosen to minimize irrelevant data, while minimizing the frequency-response delay of the filter. Typically, a small band gap introduces a phase shift of the filtered signal and makes the filtration process difficult at the edges of the dataset. (For further information about time-domain filters, see [1]). We demonstrate this in Supplementary Figure S4 on a generated sine curve. The filtered sine wave exhibits a phase-shift relative to the original signal. The first four periods of the filtered signal (to the left of the red dotted line) do not have the same amplitude as the original sine wave. The same behavior can be observed with the actual dataset in Supplementary Figure S5. For the analysis of the amplitude as a function of time, we are not concerned with the phase of the data. To reconcile the misrepresented amplitude, however, we discard the initial four oscillations of the filtered data before further analysis.

Supplementary Table S1. Parameters of the used filter and their description.

Parameter	Value	Description
First Stopband Frequency	0.5 Hz	Frequencies below this are filtered out
First Passband Frequency	0.8 Hz	Frequencies between these two values are kept
Second Passband Frequency	1.2 Hz	
Second Stopband Frequency	2.0 Hz	Frequencies above this are filtered out
First Stopband Attenuation	60 dB (*default*)	Minimum factor to divide the signal by at the first stopband frequency
Passband Ripple	1 dB (*default*)	Factor to multiply the signal by between the passband frequencies
Second Stopband Attenuation	60 dB (*default*)	Minimum factor to divide the signal by at the second stopband frequency
Sampling Frequency	10 Hz	Sampling rate of the data. Equals to the framerate of the video

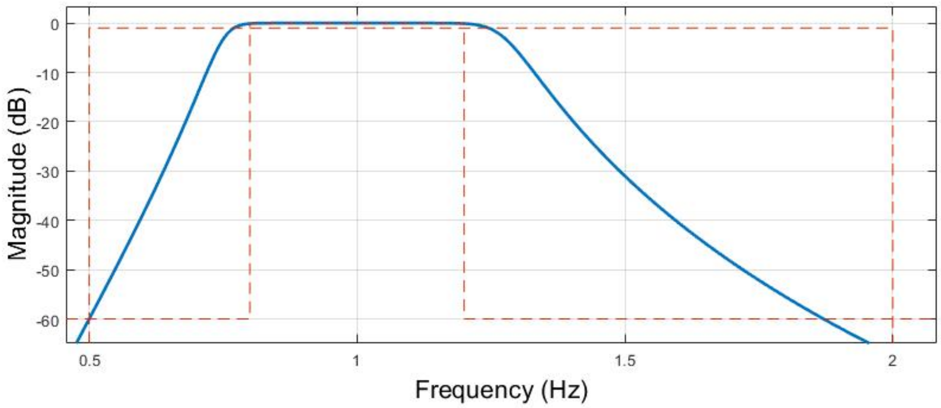

Supplementary Figure S3. Frequency response of the band-pass filter used to process the oscillatory data of probe oscillation. The inner vertical dashed lines represent the passband frequencies, indicating the stable region of the response. The outer dashed lines represent the stopband frequencies; all signal outside this region gets filtered out.

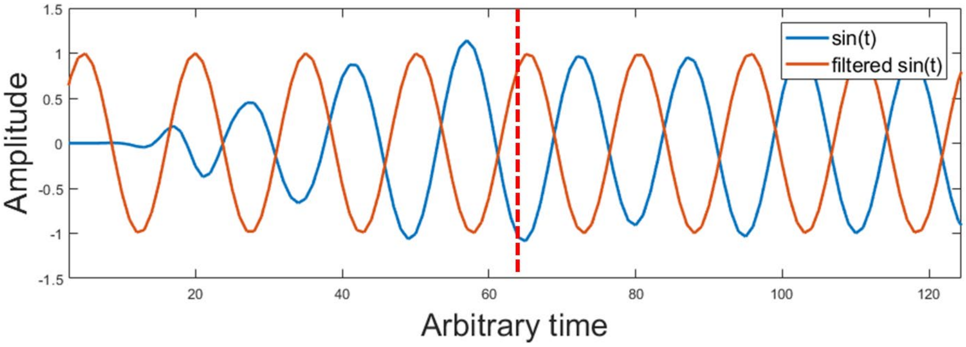

Supplementary Figure S4. Demonstration of the artifact caused by the filter at the edge of the dataset. (Orange) a generated sine wave and (blue) a filtered sine wave plotted next to each other. The filtered sine wave exhibits a phase-shift relative to the original signal. The first four periods of the filtered signal (to the left of the red dotted line) do not have the same amplitude as the original sine wave.

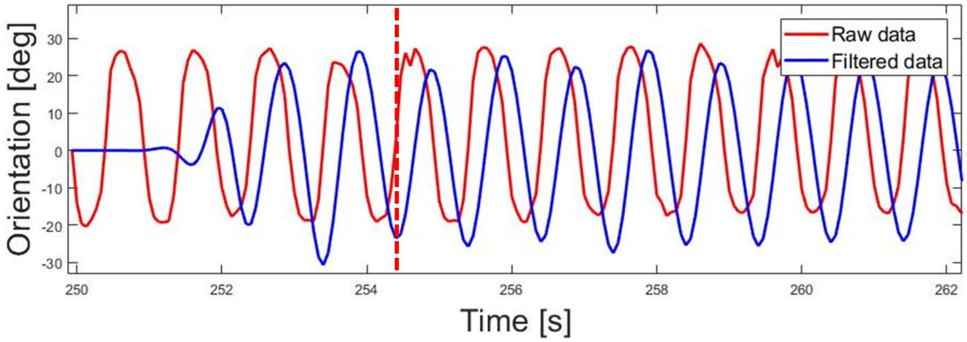

Supplementary Figure S5. The initial several oscillations of the probe. The filtered data superimposed with the raw data. Due to the frequency response of the filter, a small phase-shift is present and the first several oscillations are not adequately represented. We thus remove the first four oscillations from the dataset.

The envelope was extracted from the filtered data using the MATLAB *envelope()* function. The function returns the upper and lower envelopes of the input data as the magnitude of its analytic signal. The analytic signal is calculated using the discrete Fourier transform of the data. For more information on the *envelope()* function, refer to MATLAB documentation.

Determination of optimal dye amount for fluorescent imaging.

To determine the amount of dye to be added to the sample for optimal contrast of stained images, the procedure in [2] for *Drosophila* was modified as follows. Hemolymph was extracted via the proleg-wounding method directly into an 8-well incubation optical cell; 4–5 droplets (80–100 ul) of hemolymph were placed in each of four wells, and 5, 10, and 15 ul of 50 μg/ml of rhodamine-PNA solution in PBS buffer was added to three of the wells. One well was left without any dye as a control.

The fluorescent and phase-enhanced images of the cells and structures adhered to the substrate from all four chambers are presented in Supplementary Figure S6. A histogram stretch using the microscopy software was performed for best visualization.

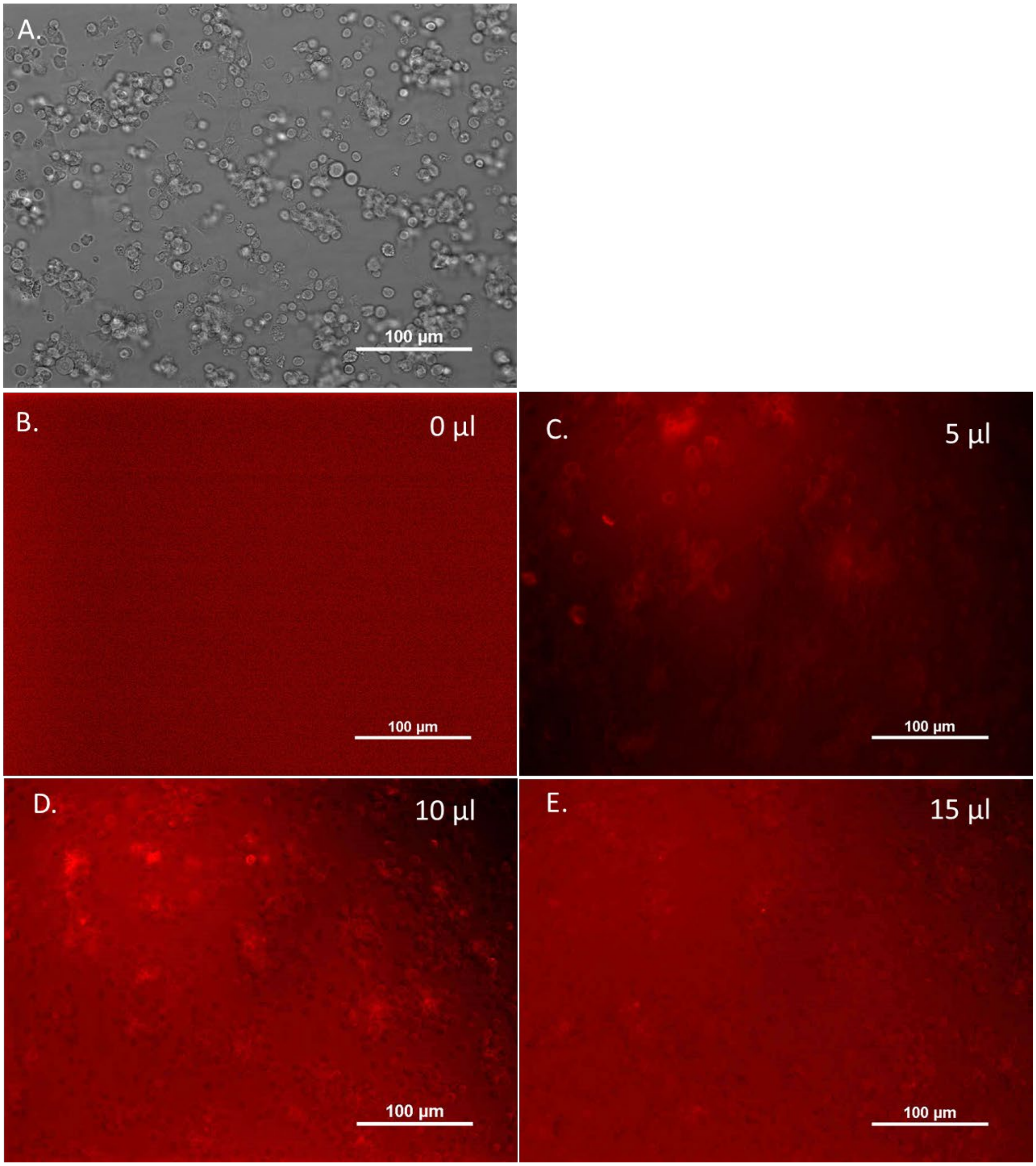

Supplementary Figure S6. Hemolymph stained and incubated for 10 minutes with PNA dye. A. Phase-enhanced and B-C fluorescent images of cells and structures adhered to the substrate dyed with 0, 5, 10, and 15 µl of PNA, respectively. The brighter red region indicates a larger dye amount and, therefore, presence of the glycosylated proteins. The brightness and contrast of the images were adjusted for the best visibility, so the red background does not necessarily represent the presence of dye. Instead, the highest brightness difference between the background and the object of interest is desired. The optimal dye volume per 100 µl of hemolymph is 5–10 µl.

The uncertainty of hemolymph volume in each sample comes from the necessity to minimize the interaction between hemolymph and foreign surfaces due to extreme adhesiveness of the hemocytes. Thus, hemolymph was bled directly from larvae into the incubation wells. Its volume could be approximated only by the number of drops that fell from the wound into each well. The volume of each drop was roughly 20 µl, and each incubation well received 4 or 5 droplets.

1 Oppenheim, A., Willsky, L. & Nawab, S. Time-and frequency characterization of signals and systems. *Signals and Systems*, 427-519 (2014).
2 Scherfer, C. *et al.* Isolation and characterization of hemolymph clotting factors in *Drosophila* melanogaster by a pullout method. *Curr. Biol.* **14**, 625-629 (2004).
